# Sympatric Speciation of Tibetan Loaches (*Triplophysa*) Driven by Dietary Niche Specialization

**DOI:** 10.1002/ece3.74001

**Published:** 2026-07-22

**Authors:** Sijia Liu, Ruoyu Zhang, Zhengli Wang, Kemao Li, Wulong Ma, Jinliang Wei, Mingzhu Wang, Wen‐Jun Ma, Shengxue Chen, Kai Zhao, Guogang Li, Fei Tian

**Affiliations:** ^1^ Key Laboratory of Adaptation and Evolution of Plateau Biota, Qinghai Provincial Key Laboratory of Animal Ecological Genomics, Northwest Institute of Plateau Biology, Chinese Academy of Sciences Xining China; ^2^ University of Chinese Academy of Sciences Beijing China; ^3^ College of Life Sciences Qinghai Normal University Xining China; ^4^ Qinghai Provincial Fisheries Technology Extension Center Xining China; ^5^ Service Center of Agricultural Husbandry and Water Conservancy Menyuan China

**Keywords:** dietary niche partition, gut microbiomes, mitochondrial genome, phylogenetics, sympatric speciation, *Triplophysa*

## Abstract

The Qinghai‐Tibeta Plateau is a hotspot for endemic fish biodiversity. Multiple isolated glacial lakes provide an ideal scenario for verifying the speciation mode of highland ichthyofauna. Sympatric speciation, a controversial topic in evolutionary biology, is driven by ecological niche differentiation, including dietary specialization. The coexistence of *Triplophysa leptosoma* and *Triplophysa longianguis* in a small semi‐enclosed postglacial Sunmcuo Lake makes it an excellent model for testing speciation mechanisms. Phylogenetic and population genetic analyses revealed monophyletic groups of *T. longianguis* and *T. leptosoma* . Together with the estimated divergence time, our results confirmed the sympatric speciation of *T. longianguis* and *T. leptosoma* . Gut contents and stable isotope analysis demonstrated the overlap of food resources with species‐specific private diet items, which is consistent with the observed morphological variations in trophic structures. The sequencing of 16S rRNA genes and LEfSe analysis revealed similar compositions and different functions of the gut microbiota between *T. longianguis* and *T. leptosoma* , which might contribute to host adaptation to specialized diets. Overall, the results of the present study identified a new case of sympatric speciation of *Triplophysa* fish and provided novel insight into sympatric speciation underlying the synergistic mechanism of the gut microbiomes and dietary specialization, which improved our understanding on the formation of endemic fish biodiversity on the Qinghai–Tibeta Plateau.

## Graphical abstract

The sympatric speciation of Tibetan loaches in a semi‐enclosed postglacial lake on the Qinghai‐Tibet Plateau, which is driven by the synergistic mechanism of the gut microbiomes and dietary specialization.

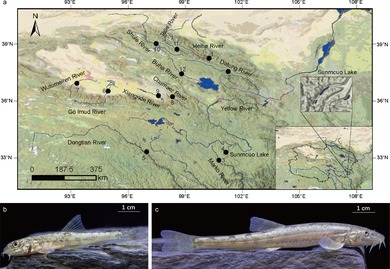

## 1 Introduction

The Qinghai–Tibet Plateau (QTP) is characterized by chronic cold, high salinity and alkalinity, and limited nutritional resources and serves as a cradle for endemic fish. The Tibetan loach *Triplophysa* Rendahl 1933 represents the primary ichthyofauna of the QTP and is widely distributed in the lakes and rivers of the QTP and surrounding regions (Chen et al. 1996; Yuan et al. 2020; Qian et al. 2023; He et al. 2024). Phylogenetic and biogeographic evidence suggests that the speciation of *Triplophysa* fishes occurred during the uplift of the QTP with a complex evolutionary history (Qian et al. 2023). To cope with food shortages, *Triplophysa* species have evolved normal‐ and scrape‐feeding behaviors with morphological diversification in the trophic apparatus, allowing them to survive in food‐restricted environments (Feng, Tang, et al. 2019). Therefore, drainages on the QTP provide natural laboratories to test speciation modes under extreme environments.

Sympatric speciation is one of the most controversial topics in evolutionary biology. It is defined as the origin of new species from a single species in the absence of geographic barriers (Elmer et al. 2010; Bird et al. 2012; Foote 2018; Richards et al. 2019). Four criteria have been proposed to approve sympatric speciation (Coyne and Orr 2004): (1) overlapping geographic distribution; (2) genetically based reproductive isolation; (3) sister taxa in phylogenetic relationships; and (4) no historical isolation. Although sympatric speciation is considered rare in nature, some compelling evidence, such as African cichlids in crater lakes (Barluenga et al. 2006), Howea palms of Lord Howe Island (Gavrilets and Vose 2007), and Darwin's finches (De León et al. 2014), has been widely regarded as the mode of speciation, which occurred under ecological conditions related to intraspecific competition for trophic resources (Barluenga et al. 2006; Bolnick and Fitzpatrick 2007; Getz et al. 2016; Sutra et al. 2019).

The divergence of a panmictic population is caused mainly by disruptive selection through ecological competition for traits associated with assortative mating (Nosil et al. 2009; Getz et al. 2016; Recuerda et al. 2023). Diet is a key biotic component of the ecological niche and plays a critical role in ecological speciation and diversification. Diet differentiation has been hypothesized to be a driving factor for divergence in sympatric populations (Grant and Grant 1979; Zhao et al. 2009; Zhang et al. 2013; Galvez et al. 2022; Pillay et al. 2025). Analysis of dietary composition (i.e., stomach contents) and trophic position (i.e., stable isotopes) revealed that coexistent cichlid fishes, with diverse lip and mouth structures, shared broad dietary niches with species‐specific private resources. These findings indicated that selection favored individuals with the ability to explore alternative resources through phenotypic specialization (Galvez et al. 2022). Therefore, the collaboration between shifts in feeding preference and variations in trophic morphology facilitated disruptive selection and ecological speciation among phylogenetically closely related species (Todd Streelman and Danley 2003; Sun et al. 2022; Neves et al. 2025).

The gut microbiomes play an important role in host adaptation to diverse nutritional resources, which expand host food resources and facilitate interspecific trophic differentiation (Greene et al. 2020; Kuang et al. 2022; Ren et al. 2025). In sympatric blind mole‐rats, the community composition of the host gut microbiota did not significantly differ, and their functional divergence was caused mainly by differences in host diet, suggesting the evolution of the gut microbiomes with dietary differentiation (Kuang et al. 2022). Studies on herbivorous and carnivorous coral reef fishes, mammals and benthic and limnetic ecotypes of three spine stickleback have demonstrated the concordance between gut microbiome shifts and host dietary specialization and have emphasized the influence of dietary selective pressures on gut microbiome assembly. These results indicate that the composition and function of gut microorganisms reflect host dietary niches and trophic morphologies and mimic host evolution and associated speciation (Rennison et al. 2019; Degregori et al. 2024). However, the role of the gut microbiota in the coexistence of fish on the QTP remains understudied.

*Triplophysa leptosoma* (Herzenstein 1888) is widely distributed on the QTP and forms a sister relationship with *Triplophysa longianguis* (Wu and Wu 1984), which is endemic to Sunmcuo Lake, a semienclosed postglacial lake connected to the Yellow River through a torrential mountain stream. Previous study on *Gymnocypris* fishes in Sunmcuo Lake linked dietary differentiation to sympatric speciation (Zhao et al. 2009; Sun et al. 2022). These findings prompted our hypothesis that *T. leptosoma* and *T. longianguis* represented another case of sympatric speciation in Sunmcuo Lake, which was driven by diet specialization synergized with the divergence of the gut microbiomes.

To test this hypothesis, we integrated mitochondrial genomes and Cyt *b* sequences to reconstruct the phylogenetic relationship and verify the speciation patterns of these two species. By combining gut content, stable isotope, and gut microbiome analyses, we aimed to infer the role of dietary differentiation in sympatric speciation and explore the influence of the gut microbiomes on dietary specialization in the two *Triplophysa* fishes. Our findings offer critical insights into the formation of ichthyofaunal biodiversity in the highland aquatic environment of the QTP.

## 2 Materials and Methods

### 2.1 Sample Collection

**FIGURE 1:**
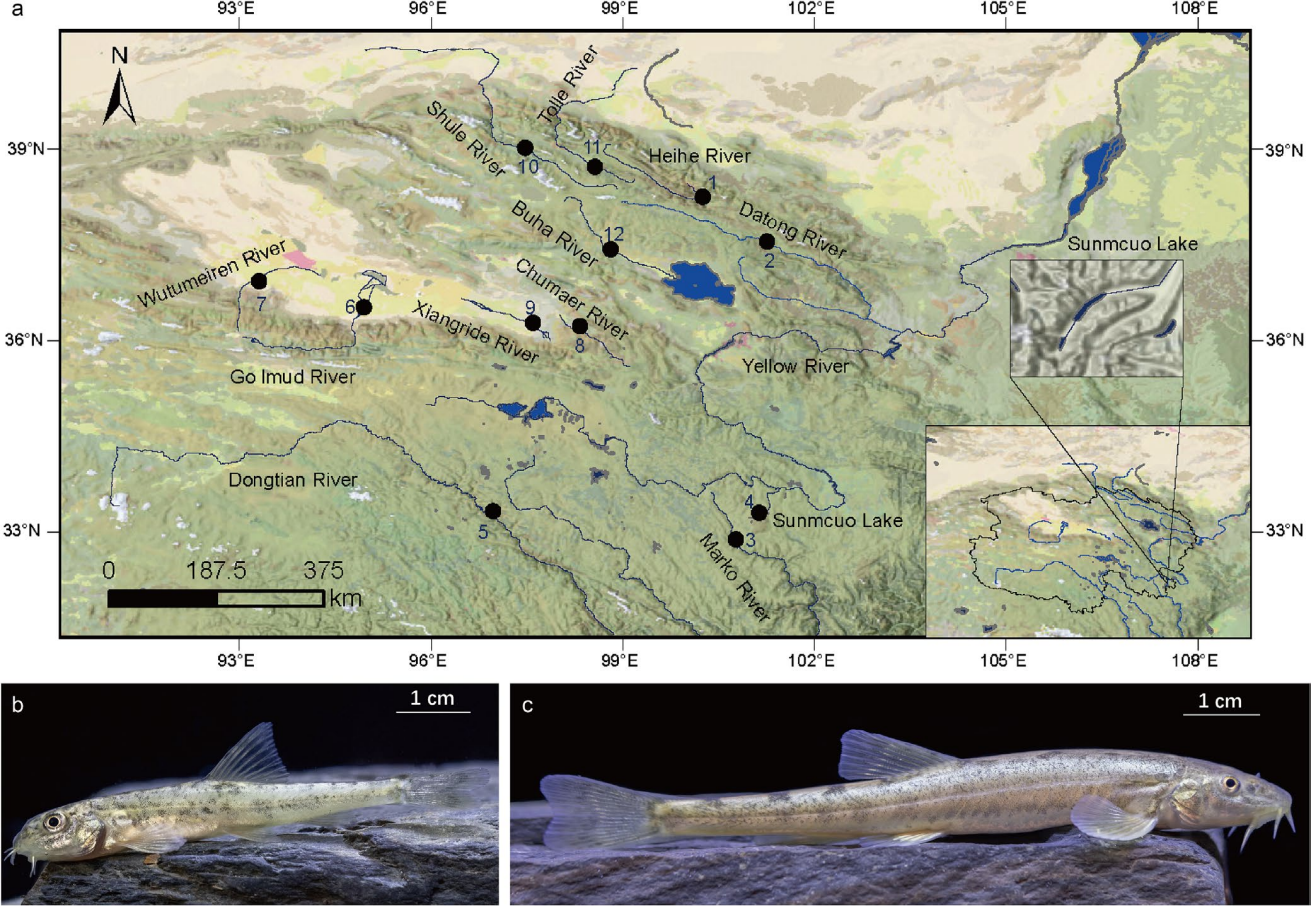
(a) Sampling locality. Black circles indicated the sampling sites. 1, 10, and 11 represented sampling sites in Hexi Basin; 2, 3 and 4 represented sampling sites in the Yellow River, Sunmcuo Lake and the Yangtze River; 6–9 represented sampling sites in the Chaidamu River; 12 represented a sampling site in Lake Qinghai Basin. (b) *Triplophysa longianguis* . (c) *Triplophysa leptosoma* . Both photos were taken by Dr. Sijia Liu.

Field investigations were performed from August 2021–2023 in Sunmcuo Lake, the Yellow River, the Yangtze River, the Heihe River, and the Chaidamu River systems on the QTP (Figure 1 and Table S1), which were approved and supervised by the Qinghai Provincial Bureau of Fishery. In total, 213 samples from *T. leptosoma* and *T. longianguis* were collected from the sampling area and recognized based on the description by Wu and Wu (1992) and He et al. (He et al. 2024). The dorsal fin of each sample was collected and stored at −80°C for DNA extraction.

In addition, 24 samples of each species were transported to the laboratory and sprayed with 70% ethanol for sterilization. The fish were aseptically dissected, and the gut contents were gently squeezed from the intestine, which was disinfected with 70% ethanol and sterile PBS to remove transient bacteria. The gut contents were collected from 24 specimens of each species, of which 10 were subjected to microscopic examination and 14 were subjected to microbiome sequencing. Muscles of 10 samples from each species were collected from each species for isotope analysis. All animal procedures were approved by the Animal Care and Use Committee of the Northwest Institute of Plateau Biology, Chinese Academy of Sciences (Approval No. NWIPB‐202006).

### 2.2 DNA Extraction and Mitochondrial Genome Sequencing

Total DNA for mitochondrial genome sequencing and population analysis was extracted from the dorsal fin using the Tissue Blood DNA Extraction Kit (Tiangen, Beijing, China). The integrity of the resulting total DNA was tested via 1% agarose gel electrophoresis, and the purity and concentration were detected by an ultramicro spectrophotometer, Nanodrop NC2000 (Thermo, USA), and subsequently stored in liquid nitrogen for subsequent analysis.

The extracted DNA from *T. leptosoma* and *T. longianguis* was broken by sonication into approximately 350 bp fragments, followed by end–repair and the addition of a sequencing adapter. PCR amplification was conducted, and the products were subsequently purified via magnetic beads for library construction. The sequencing library was checked on an Agilent Bioanalyzer using an Agilent High Sensitivity DNA Kit (Agilent Technologies, USA) and then quantified on a Promega QuantiFluor fluorescence quantification system (Promega, USA) using the Quant‐iT PicoGreen dsDNA Assay Kit (Thermo Fisher, USA). The qualified libraries were sequenced on the Illumina NovaSeq 6000 sequencing platform (Illumina, USA) at Personalbio Technology Co. Ltd. (Shanghai, China).

### 2.3 Mitochondrial Assembly and Phylogenetic Analysis

The raw data were processed for quality control using fastp (v0.20.0) to remove adapters and low‐quality reads. High‐quality reads were assembled into the complete mitochondrial genome using SPAdes (v3.10.1), which was annotated using Mitos2 (http://mitos2.bioinf.uni‐leipzig.de/index.py) (Bernt et al. 2013). The annotation results were further manually corrected according to published mitogenomes of *Triplophysa* fishes (Table S2). The secondary structures of tRNA were obtained from the annotation outputs of Mitos2. The mitochondrial genome map was generated using OGDRAW (https://chlorobox.mpimp‐golm.mpg.de/OGDraw.html) (Greiner et al. 2019). The base composition and amino acid contents were calculated using MEGA 11 software (Tamura et al. 2021), including A + T content, AT skew, and GC skew. Relative synonymous codon usage (RSCU) was analyzed using the RSCU module implemented in PhyloSuite (version 1.2.3) (Zhang et al. 2020).

The complete mitochondrial genome of *Triplophysa* fishes was downloaded from a public database (Table S2), which was imported into PhyloSuite (version 1.2.3) (Zhang et al. 2020). MAFFT was used for sequence alignment, and MODELFINDER was subsequently used to select the best‐fit model. The phylogenetic tree was constructed using the maximum likelihood (ML) method and Bayesian inference (BI), which was illustrated via iTOL (version 6).

### 2.4 Population Genetic Analysis

The mitochondrial gene Cyt *b* was selected for PCR amplification using the universal primers L14724 (5′–GACTTGAAAAACCACCGTTG–3′) and H15915 (5′–CTCCGATCTCCGGATTACAAGAC–3′) (Xiao et al. 2001; Li et al. 2008). The PCR products were examined by electrophoresis and sent to General Biologicals (Anhui) Co. for Sanger sequencing on an Agilent 3730 platform. The sequences were assembled using SeqMan (DNASTAR, Madison, WI, USA); sequence alignment was performed via CLUSTALW embedded in MEGA X, and corrections were made by manual inspection (Thompson et al. 1994). DnaSP (version 5.1) was used to calculate the genetic diversity of the population (Librado and Rozas 2009), including the number of polymorphic sites (S), the number of haplotypes (H), haplotype diversity (Hd), and nucleotide diversity (Pi). Haplotypes of Cyt *b* sequences (Table S3) were used to construct the phylogenetic tree via PhyloSuite (version 1.2.1), with the same method used for phylogenetic analysis of the mitochondrial genome PhyloSuite (version 1.2.1). According to the lineages in the phylogenetic tree, K2P genetic distance and genetic differentiation (*F*_st_) were calculated using MEGA X and DnaSP (version 5.1).

### 2.5 Molecular Dating

The divergence time was estimated based on the Cyt *b* dataset using BEAST (version 1.10.4) (Suchard et al. 2018). The selected substitution model was HKY + I + G, with a lognormal uncorrelated relaxed clock model, combined with a Yule process tree prior and a piecewise linear with constant root population size prior. Given that no fossil record of *Triplophysa* fishes was available, two validated time calibrations were employed, including the split between *Sinibotia superciliaris* and *Leptobotia zebra* at 13.9 Ma (Kumar et al. 2022) and the estimated separation of *Triplophysa bleekeri* and *Triplophysa leptosoma* at 7.9 Ma (Feng et al. 2017). Four independent Markov chain Monte Carlo (MCMC) runs were conducted for 200 million generations each, with sampling every 1000 steps. The first 20% of the samples were discarded as burn‐in, and the remaining samples were combined using LogCombiner 1.10.4 in BEAST 1.10.4 (Suchard et al. 2018). Convergence was assessed by an average standard deviation of split frequencies < 0.01 and ESS values > 200 in Tracer v1.7.1 (Rambaut et al. 2018). The maximum clade credibility tree was constructed using the TreeAnnotator 1.10.4 module of BEAST 1.10.4 (Suchard et al. 2018) and edited using Figtree v1.4.3.

### 2.6 Carbon and Nitrogen Isotope Analysis

To determine the position of the trophic level and assess the feeding habits, carbon and nitrogen isotopes were determined in *Triplophysa leptosoma* and *T. longianguis* of Sunmcuo Lake. Dorsal muscle of 1 cm^3^ in size was cut from 10 samples of each species and dried in an oven at 55°C for 48 h until a constant weight was reached. The samples were ground into powder in a high‐throughput tissue grinder. Powder (0.6–1.0 mg) was coated with aluminum foil, and an elemental analyzer (Sercon Integra 2, UK) was used. IAEA‐600 caffeine was used as a standard (Letourneur et al. 2013). Carbon and nitrogen stable isotope analyses were carried out at Shanghai Paisenow Biotechnology Co. Ltd. Each sample was assayed three times and averaged to ensure the accuracy of the results. For the representation of carbon stable isotope abundance, we used the δ value (delta value), which is a widely accepted representation. Specifically, the δ value reflects the difference in isotope ratios between the sample and the laboratory reference material (i.e., the working standard) in terms of the difference in parts per thousand (‰, per mill). In the measurement, the difference in isotope ratios between the sample and the working standard is first calculated, and this difference is subsequently converted to a δ value relative to the international isotope standard to obtain the final representation of carbon stable isotope abundance.

$$\delta =\frac{\mathrm{RSA}-\mathrm{RST}}{\mathrm{RST}}\times 1000=\left(\frac{\mathrm{RSA}}{\mathrm{RST}}-1\right)\times 1000$$

where δ represents ^13^C or ^15^N and R_SA_ and R_ST_ are the isotope ratios (^13^C/^12^C or ^15^N/^14^N) of the measured sample and the reference standard sample, respectively. The error of sample analysis was within ±0.3‰. Student's *t*‐test was applied to compare δ^13^C or δ^15^N values between two species, with a *p* value of 0.05 set as the significance level.

### 2.7 Examination of Intestinal Contents

The intestines were collected from 10 samples of each *Triplophysa* species from Sunmcuo Lake and then were tied tightly at both ends to fix in 4% PFA for 24 h. The intestinal contents were rinsed in purified water three times and then placed in Petri dishes for stereoscopy examination (Nikon, Japan). Based on the morphological characteristics, the food items were identified as plant material and seeds, periphytic algae, insect pieces and larvae, and zoobenthos, which were weighed to determine their relative abundance (Figure S1). Unrecognized materials (less than 10% of total weight) were excluded from the diet analysis.

### 2.8 DNA Extraction and 16S rRNA Gene Sequencing

For 16S rRNA sequencing, DNA was extracted from the intestine using Mag Beads Fast DNA Kit for Soil (MP Biomedicals, CA, USA). The quality and quantity of the DNA were measured using 0.8% agarose gel electrophoresis with a Nanodrop NC2000 (Thermo, USA). The gut microbiomes of 14 samples of each species were investigated using 16S rRNA gene sequencing. The V3‐V4 region of bacteria was amplified via PCR using universal primers (Table S4). PCR with a negative control was performed, and the products were purified for library construction. PCR amplicons were quantified using Quant‐iT PicoGreen dsDNA Assay Kit (Thermo Fisher, USA) in a microplate reader (BioTek, FLx800). According to the manufacturer's protocol, the sequencing library was constructed using the TruSeqIM DNA Sample Prep Kit. After end‐repair, adapters were ligated with A bases, which were then purified using BECKMAN AMPure XP beads (BECKMAN, USA). After size selection, the quality and quantity of the sequencing library were determined using Agilent Bioanalyzer 2100 (Agilent, USA) and Promega QuantiFluor (Promega, USA). The constructed library was subsequently sequenced on the Illumina MiSeq PE300 platform (Illumina, USA) by Personalbio Technology Co. Ltd. (Shanghai, China).

### 2.9 Data Processing

The raw sequencing data were subjected to removal of sequencing adaptors and low‐quality reads by fastp (0.19.6) and then imported into Vsearch (v2.13.4) (Rognes et al. 2016) for OUT clustering and QIIME 22019.4 (Bolyen et al. 2019) for microbiome bioinformatics. Briefly, sequencing data were demultiplexed, followed by trimming of primers using the cutadapt plugin (Martin 2011). The sequences were then merged, filtered and dereplicated, and all the unique sequences were clustered at 98% followed by chimera removal. The non‐chimera sequences were re‐clustered at 97% to generate the OTU sequences and the OTU table. The representative sequences were aligned using mafft (Katoh et al. 2002) for phylogenetic tree construction using fasttree2 (Price et al. 2009). Taxonomy was assigned to the OTUs using the classify‐sklearn naïve Bayes taxonomy classifier in the feature‐classifier plugin (Bokulich et al. 2018) against Silva v132 at 99% of the OTU reference sequences (Quast et al. 2013). The microbial community of each sample was classified and recorded at the phylum, class, order, family, genus, and species levels. The alpha diversity of the gut microbiomes of the two species was evaluated based on the Shannon index, Simpson index, species richness index and Pielou index. The Mann–Whitney *U* test was applied to compare the differences in α diversity between the two species. Bray‐Curtis distances matrix was constructed using the adonis2 function in the vegan package (Dixon 2003) in R (v 4.4.2), which was applied to group difference comparisons using permutational multivariate ANOVA (PERMANOVA). The result was visualized using principal coordinates analysis (PCoA) with the ape package (Emmanuel et al. 2018) in R (v 4.4.2). Linear discriminant analysis effect size (LEfSe) was carried out to examine the variations in the gut microbiome between the two species (Segata et al. 2011). Taxa with absolute LDA scores > 4 were retained (Yang et al. 2025). The alterations in functional pathways were assessed based on the KEGG database for bacterial taxa via 16S rRNA sequencing (Langille et al. 2013). The neutral process in bacterial assembly was evaluated using the Sloan model in the R v 3.5.1 code (Sloan et al. 2006; Chen et al. 2019).

## 3 Results

### 3.1 Mitochondrial Genomes of *T. leptosoma* and *T. longianguis*

**FIGURE 2:**
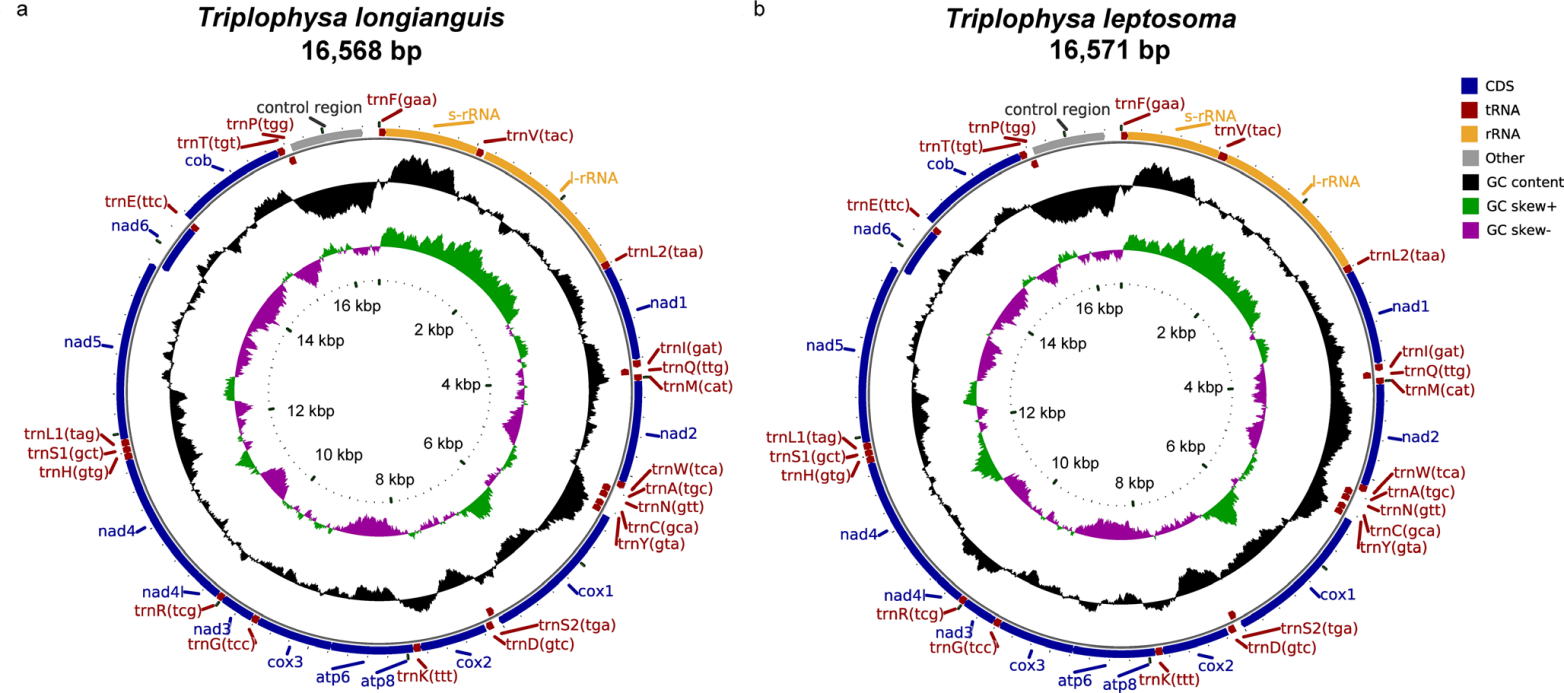
Mitochondrial genome structures of *Triplophysa longianguis* (a) and *Triplophysa leptosoma* (b). Blue blocks: CDSs of mitochondrial genes; red blocks: Transfer RNAs; yellow blocks: Ribosomal RNA; gray blocks: Others; black bars: GC contents; green bars: GC skew +; purple: GC skew –.

The mitochondrial genomes (mitogenomes) of *T. leptosoma* and *T. longianguis* from Sunmcuo Lake were 16,565 and 16,571 bp in length, with GC contents of 43.02% and 42.61%, respectively (Table 1). Like *Triplophysa* species, the double‐stranded mitogenome contains 37 genes, including 13 protein‐coding genes (PCGs), 22 tRNA genes and two rRNA genes, among which NAD6 and 8 tRNAs (tRNA^Gln^, tRNA^Ala^, tRNA^Asn^, tRNA^Cys^, tRNA^Tyr^, tRNA^Ser^, tRNA^Glu^, and tRNA^Pro^) are located in the light chain, and the rest of the genes are in the heavy chain (Figure 2).

**TABLE 1:** Statistics of the mitochondrial genomes of *Triplophysa leptosoma* and *Triplophysa longianguis* .

*T. leptosoma*								*T. longianguis*						
Region	Length (bp)	A%	C%	G%	T%	A + T%	G + C%	Length (bp)	A%	C%	G%	T%	A + T%	G + C%
Whole genome	16,571	28.65	25.1	17.51	28.74	57.39	42.61	16,565	28.17	25.23	17.79	28.81	56.98	43.02
atp6	684	26.32	25.88	13.74	34.06	60.38	39.62	684	29.61	25.92	23.29	21.18	50.79	49.21
atp8	168	33.33	26.79	13.1	26.79	60.12	39.88	168	34.82	21.61	21.12	22.45	57.27	42.73
cob	1141	26.38	27.43	16.3	29.89	56.27	43.73	1141	23.79	27.18	18.56	30.46	54.26	45.74
cox1	1551	25.34	24.76	18.76	31.14	56.48	43.52	1551	26.51	27.56	16.56	29.38	55.89	44.11
cox2	691	28.51	24.6	17.66	29.23	57.74	42.26	691	19.35	25.81	35.48	19.35	38.71	61.29
cox3	784	27.3	25.38	16.96	30.36	57.65	42.35	784	25.34	24.82	18.57	31.27	56.61	43.39
nad1	975	26.05	26.56	16.51	30.87	56.92	43.08	975	28.65	25.18	17.22	28.94	57.6	42.4
nad2	1045	27.08	28.23	16.17	28.52	55.6	44.4	1045	33.33	27.38	13.69	25.6	58.93	41.07
nad3	349	26.07	24.07	16.05	33.81	59.89	40.11	349	26.61	25.58	13.89	33.92	60.53	39.47
nad4	1382	27.71	24.46	15.92	31.91	59.62	40.38	1382	26.66	25.26	17.47	30.61	57.27	42.73
nad4l	297	23.91	27.95	16.84	31.31	55.22	44.78	297	24.64	25.21	17.19	32.95	57.59	42.41
nad5	1839	27.95	25.07	15.39	31.59	59.54	40.46	1839	22.9	27.61	17.85	31.65	54.55	45.45
nad6	522	17.82	15.9	30.46	35.82	53.64	46.36	522	27.5	25.11	16.06	31.33	58.83	41.17
OH	743	30.69	21.8	14.27	33.24	63.93	36.07	743	26.26	25.83	16.37	31.54	57.8	42.2
OL	31	19.35	25.81	35.48	19.35	38.71	61.29	31	18.39	15.52	29.69	36.4	54.79	45.21
rrnL	1658	34.92	21.65	21.11	22.32	57.24	42.76	1657	25.77	27.52	16.65	30.06	55.83	44.17
rrnS	952	29.58	25.68	23.47	21.26	50.84	49.16	949	30.82	21.4	14.13	33.65	64.47	35.53

**FIGURE 3:**
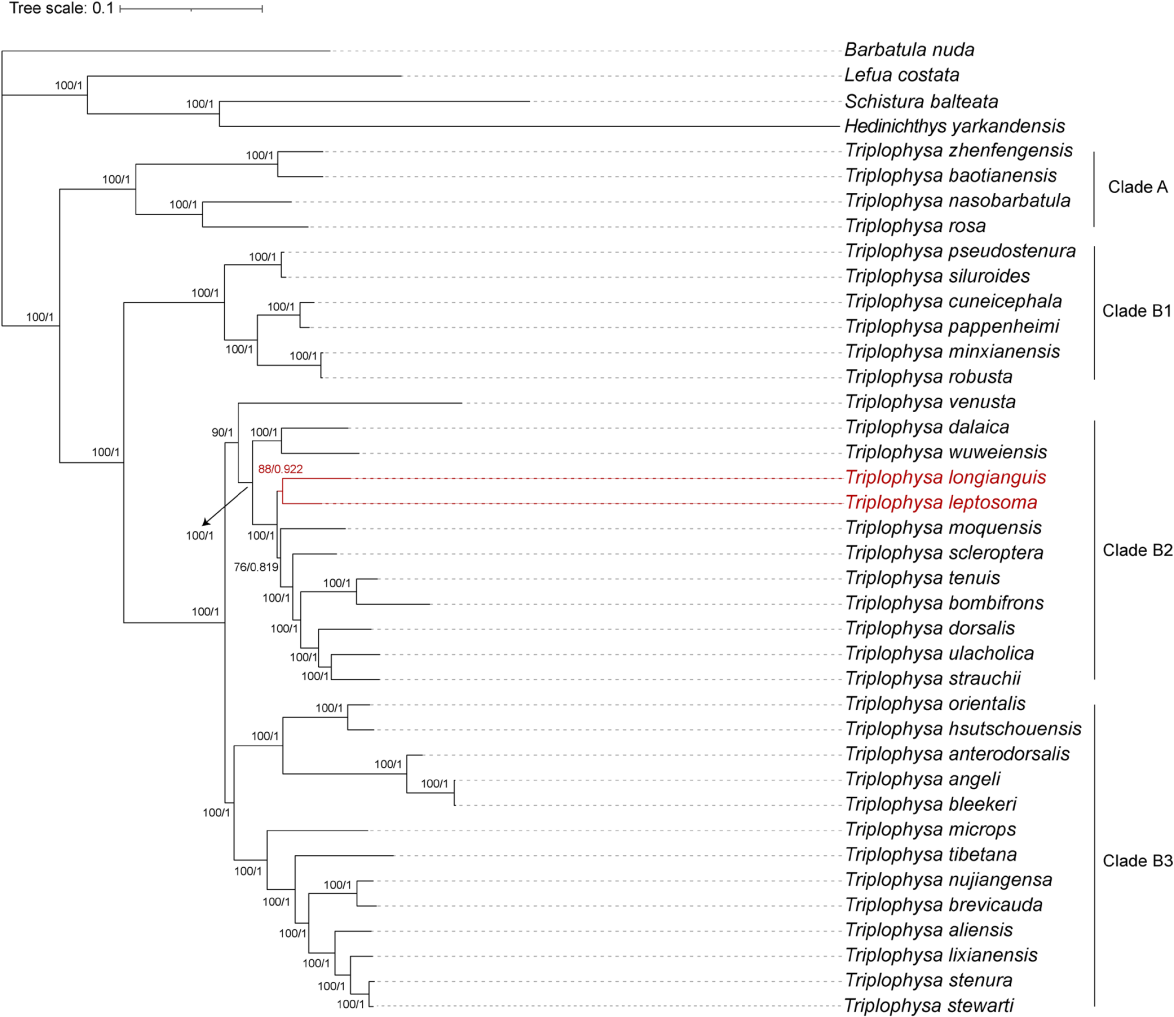
Phylogenetic positions of *Triplophysa longianguis* and *Triplophysa leptosoma* in the genus *Triplophysa*. ML and BI trees were constructed based on the sequences of the mitochondrial genomes, which demonstrated the same topology. The posterior probabilities and Bayesian support values are labeled on the branches. *Triplophysa longianguis* and *Triplophysa leptosoma* are indicated in red.

Based on the mitogenomes of 35 *Triplophysa* species and outgroups ( *Barbatula nuda* , *Lefua costata* , *Schistura balteata* and *Hedinichthys yarkandensis*), the reconstructed phylogenetic tree classified *Triplophysa* fishes into three major clades (A, B and C), which to a certain extent reflected their geographic patterns (Figure 3). The cave‐dwelling loaches clustered in Clade A, which included *Triplophysa rosa* , *Triplophysa nasobarbatula* , *Triplophysa baotianensis*, and *Triplophysa zhenfengensis* , preliminarily on the southwestern QTP. Clade B1 included *Triplophysa* species mainly from the Yellow River and the Hexi River system at the northeast edge of the QTP. Clade C1 included mainly *Triplophysa* fish in the Tarim River, Yellow River, and Lake Qinghai basin. Furthermore, *T. longianguis* and *T. leptosoma* were grouped together with the closest phylogenetic relationship in Clade C1. In Clade C2, *Triplophysa* species on the central QTP, such as the Yarlung Zangbo River and lakes from Tibet, were classified as widespread species, such as *Triplophysa stenura* and *Triplophysa orentalis*.

### 3.2 Phylogenetic Analysis of *T. leptosoma and T. longianguis* in Sunmcuo Lake

**FIGURE 4:**
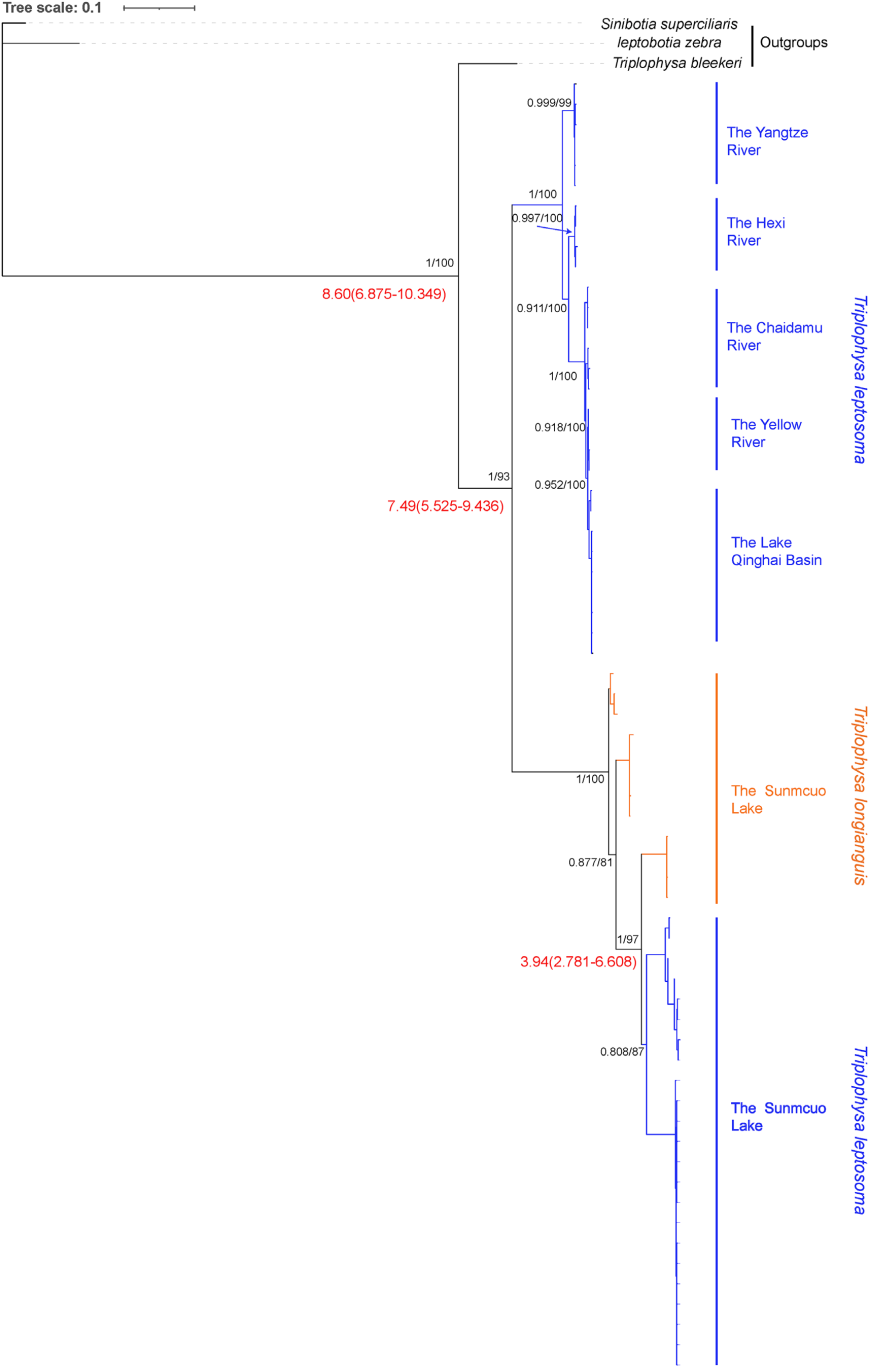
Phylogenetic tree of *Triplophysa longianguis* and *Triplophysa leptosoma* . ML and BI trees were constructed based on Cyt *b* sequences. The posterior probabilities and Bayesian support values are in black. The estimations of the divergence times and 95% HPD are labeled in red on the branches.

In total, we sequenced the Cyt *b* gene in 15 individuals of *T. longianguis* and 154 samples of *T. leptosoma* from Sunmcuo Lake, which generated 11 and 23 haplotypes, respectively. Additionally, 44 samples of *T. leptosoma* from the Yellow River, Lake Qinghai Basin, Changjiang River, Chaidamu River, and Heixi River yielded 25 haplotypes. By setting *Leptobotia zebra* and *Sinibotia superciliaris* as the outgroup, we constructed BI and ML phylogenetic trees based on 59 haplotypes from *T. longianguis* and *T. leptosoma* , which consisted of two clades. The first clade consisted of three lineages, including a lineage of *T. leptosomas* from the Yangtze River, a lineage of *T. leptosomas* from the Chaidamu River, and a lineage of *T. leptosomas* from the Hexi River, Lake Qinghai River, and Yellow River. The other clade included samples of *T. longianguis* and *T. leptosoma* from Sunmcuo Lake, which were grouped together in a paraphyletic relationship with *T. leptosomas* from other river systems (Figure 4). This phylogeny revealed that the monophyletic groups of *T. longianguis* and *T. leptosoma* in Sunmcuo Lake presented the closest genetic relationships. The estimated divergence time was 7.49 Mya (95% HPD = 5.525–9.436 Mya) between *Triplophysa* fish from Sunmcuo Lake and *T. leptosoma* from other water systems in the other clade. Additionally, the separation time of *T. longianguis* and *T. leptosoma* in Sunmcuo Lake was estimated to be 3.94 Mya (95% HPD = 2.781–6.608 Mya) (Figure 4). These results indicated that *T. longianguis* had a closer genetic relationship with *T. leptosomas* from Sunmcuo Lake than with those from other geographic populations.

### 3.3 Population Genetics of *T. leptosoma and T. longianguis* in Sunmcuo Lake

Population genetics analysis revealed that the nucleotide diversity (Pi) was 0.0429 and 0.0088 and that the haplotype diversity (Hd) was 0.933 and 0.678 in *T. longianguis* and *T. leptosoma* in Sunmcuo Lake, respectively. Additionally, the nucleotide diversity (Pi) and haplotype diversity of *T. leptosoma* from water systems outside of Sunmcuo Lake were 0.0224 and 0.961, respectively (Table 2). The genetic distance and genetic differentiation between *T. longianguis* (Sunmcuo Lake) and *T. leptosoma* (Sunmcuo Lake) reached 7.762% and 0.80323, respectively, which were lower than those among geographic populations of *T. leptosoma* , ranging from 15% to 16% and 0.93–0.94, respectively (Table 3). Consistent with the phylogenetic tree, the lower genetic differentiation and genetic distance between *T. longianguis* and *T. leptosoma* in Sunmcuo Lake suggest that their genetic relationships are closer than those among geographic populations of *T. leptosoma* .

**TABLE 2:** Population genetics of *Triplophysa* fish in Sunmcuo Lake.

Species	Sequence number	Index	Value
*T. leptosoma* (the Sunmcuo Lake)	154	S	103
		H	23
		Hd	0.678
		Pi	0.0088
*T. longianguis* (the Sunmcuo Lake)	15	S	111
		H	11
		Hd	0.933
		Pi	0.0429
*T. leptosoma* (out of the Sunmcuo Lake)	44	S	94
		H	25
		Hd	0.961
		Pi	0.0224

Abbreviations: H, number of haplotypes; Hd, haplotype diversity; Pi, nucleotide diversity; S, number of polymorphic sites.

**TABLE 3:** K2P genetic distance (%) and genetic differentiation (*F*_st_) among *Triplophysa* fish populations.

	*T. leptosoma* (the Yangtze River)	*T. leptosoma* (The Hexi River)	*T. leptosoma* (The Yellow River)	*T. leptosoma* (the Lake Qinghai Basin)	*T. leptosoma* (The Chaidamu River)	*T. leptosoma* (the Sunmcuo Lake)	*T. longianguis* (the Sunmcuo Lake)
*T. leptosoma* (the Yangtze River)		0.88001	0.89997	0.95012	0.89460	0.94042	0.81361
*T. leptosoma* (The Hexi River)	3.850		0.86028	0.94882	0.84554	0.93769	0.74333
*T. leptosoma* (The Yellow River)	4.744	3.959		0.55668	0.49043	0.94021	0.82107
*T. leptosoma* (the Lake Qinghai Basin)	4.554	3.734	0.665		0.76060	0.94281	0.84137
*T. leptosoma* (The Chaidamu River)	4.698	3.947	1.061	1.208		0.93966	0.80105
*T. leptosoma* (the Sunmcuo Lake)	16.202	15.980	16.196	16.071	16.260		0.80323
*T. longianguis* (the Sunmcuo Lake)	17.341	16.148	17.201	16.960	17.098	7.762	

*Note:* The lower left diagonal represents the K2P genetic distance (%) between two populations, and the upper right diagonal represents the degree of genetic differentiation.

### 3.4 Dietary Analysis of *T. leptosoma* and *T. longianguis*

**FIGURE 5:**
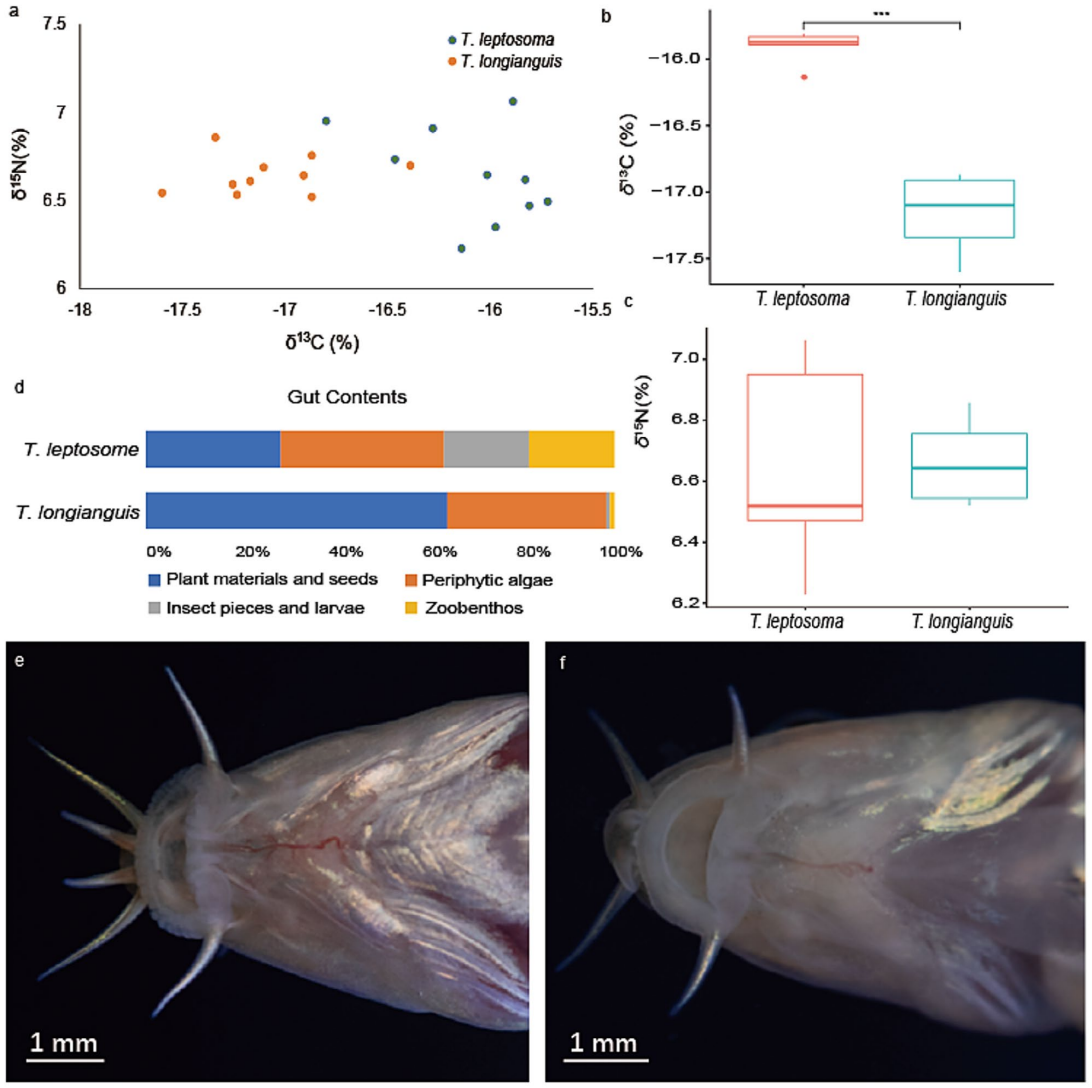
(a) Scatterplot of δ^13^C and δ^15^N isotopic values for *Triplophysa longianguis* (*n* = 10) and *Triplophysa leptosoma* (*n* = 10). Box plots of δ^13^C isotope values (b) and δ^15^N isotope values (c) for *T. longianguis* and *T. leptosoma* . Student's *t*‐test was applied to compare δ^13^C or δ^15^N values between the two species. ***p value < 0.005 in Figure 5b. (d) Profiles of the gut contents of *T. longianguis* and *T. leptosoma* . The color and length of each bar represent different dietary components and average proportions of food items. Lip structures of *T. leptosoma* (e) and *T. longianguis* (f).

It has been reported that closely related species are partitioned in resource usage to avoid competition, including food utilization. Therefore, we performed carbon and nitrogen isotope (*δ* ^13^C and *δ* ^15^N) analyses to explore the diet compositions of *T. longianguis* and *T. leptosoma* from Sunmcuo Lake, which revealed the separation of the two species in terms of diet preference (Figure 5a). No difference was detected in the stable N isotope (*δ* ^15^N) values, suggesting that *T. longianguis* and *T. leptosoma* have the same trophic levels. The relatively small stable C isotope (*δ* ^13^C) signature indicated that *T. longianguis* is a pelagic species that prefers to inhabit open water and forage with phytoplankton, whereas *T. leptosoma*, with relatively large δ^13^C values, tends to ingest benthic organisms (Figure 5b,c). Consistent with the isotope analysis results, plants and algae, as the major dietary components, accounted for 98% and 63.65% of the gut contents of *T. longianguis* and *T. leptosoma* , respectively (Figure 5d). Insects and zoobenthos were rarely identified in *T. longianguis* but accounted for 18.09% and 18.26%, respectively, of *T. leptosoma* (Figure 5d). Similarly, morphological differences in mouth structure were detected between *T. longianguis* and *T. leptosoma* in Sunmcuo Lake. The lips of *T. leptosoma* are wrinkled or milky, whereas smooth lips are observed in *T. longianguis* , which might reflect differences in feeding habits (Figure 5e,f).

### 3.5 Intestinal Microbiomes of *T. leptosoma* and *T. longianguis*

**FIGURE 6:**
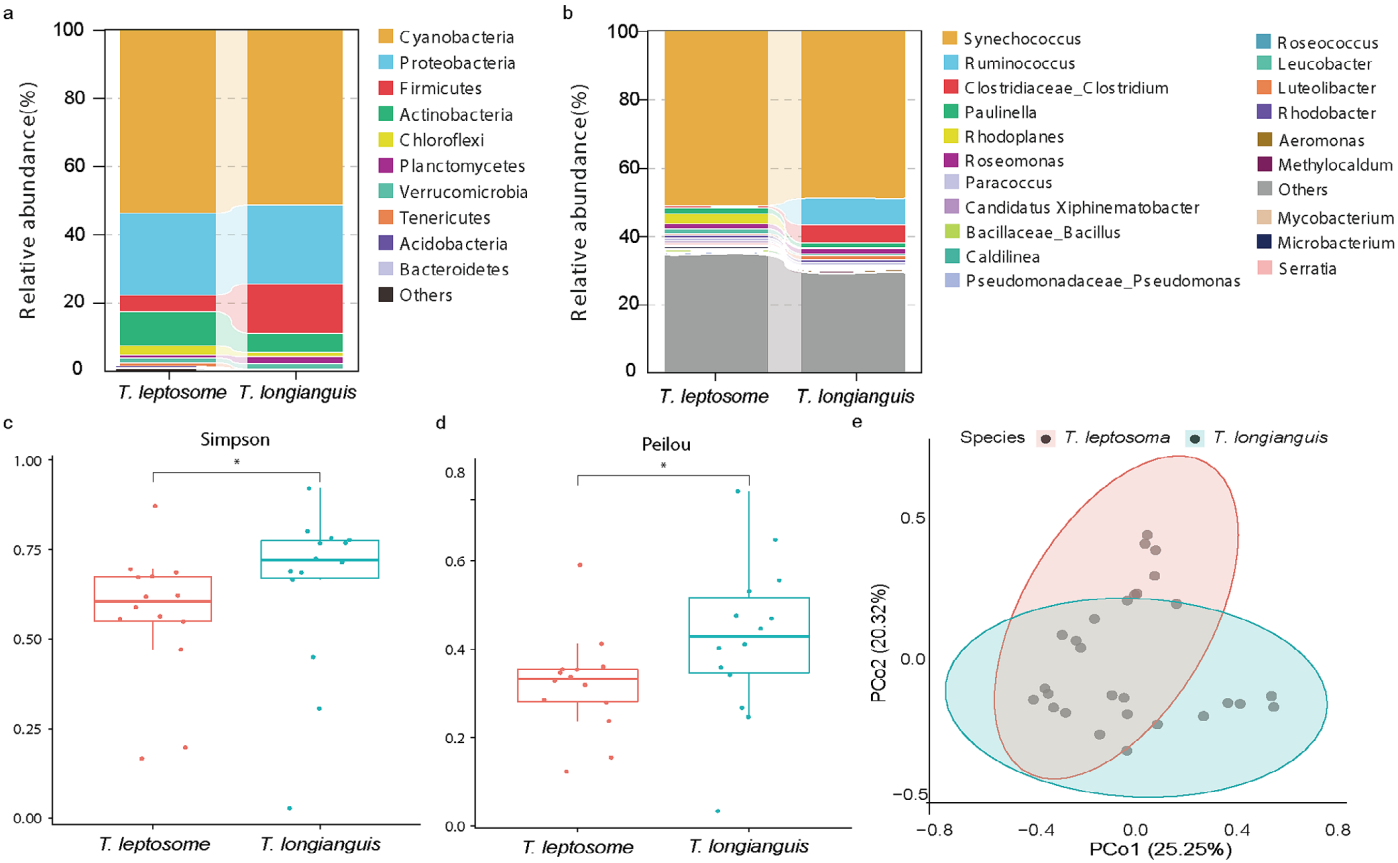
Composition of the gut microbiota at the phylum (a) and genus (b) levels in *T. longianguis* (*n* = 14) and *T. leptosoma* (*n* = 14). Box plots of the Simpson (c) and Peilou (d) indices of the gut microbiomes of *T. longianguis* and *T. leptosoma* . The Mann–Whitney *U* test was applied to compare the differences in Simpson and Peilou indices between the two species. *p value < 0.05 in (c) and (d). (e) Bray–Curtis distance of PCoA representing the calculated distance between *T. longianguis* and *T. leptosoma* (permutational multivariate analysis of variance, PERMANOVA, *p* < 0.05).

**FIGURE 7:**
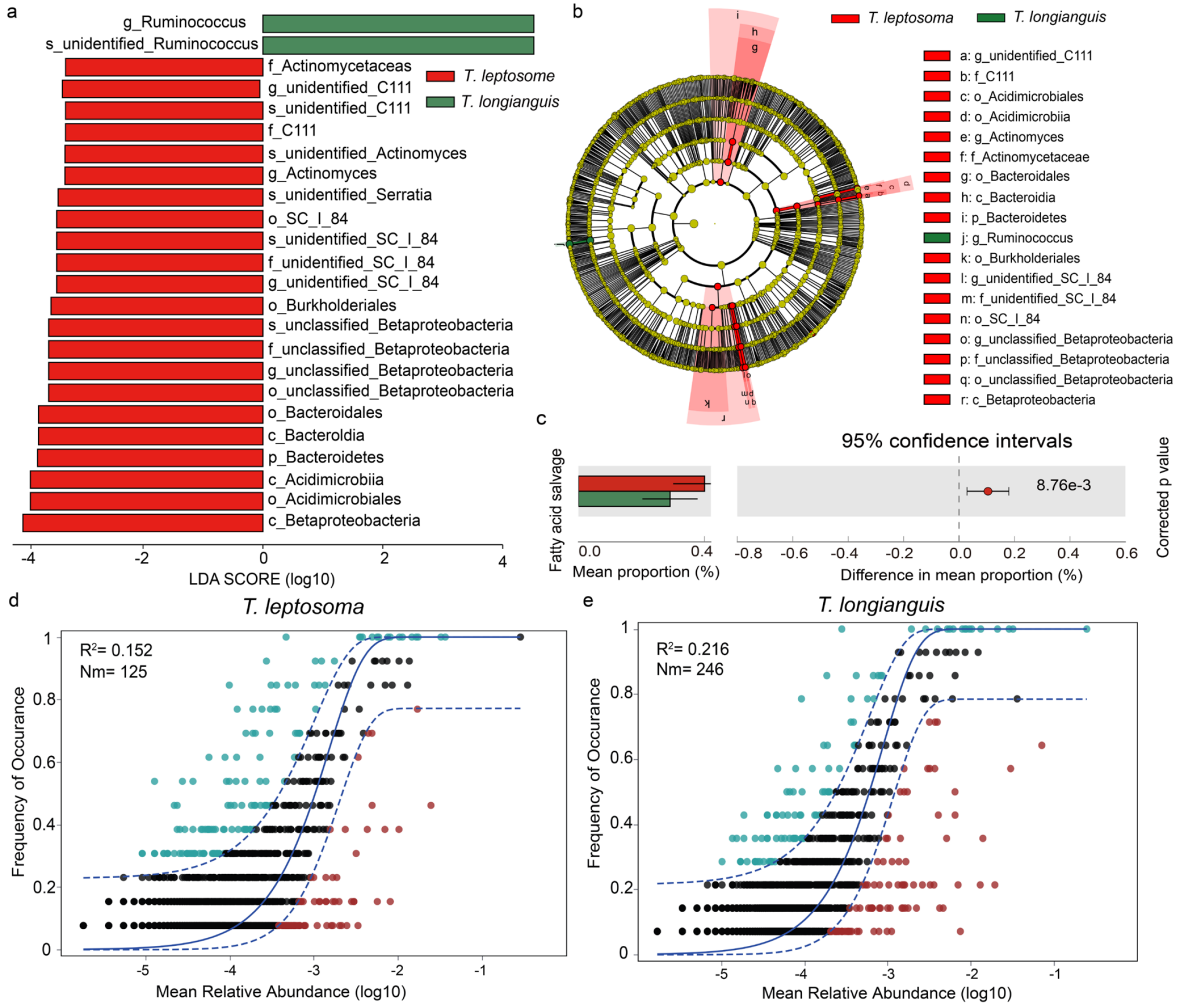
(a) Identification of significantly different taxa between the gut microbiota of *Triplophysa longianguis* and *Triplophysa leptosoma* from the phylum to the genus level via linear discriminant analysis effect size (LEfSe). (b) Cladogram of the phylogenetic position of the gut microbial lineages of *T. longianguis* and *T. leptosoma* . Each circle represents a classification (from the inner to outer rings, genus, family, order, class and phylum). (c) KEGG pathways significantly different between the gut microbiota of *T. longianguis* and *T. leptosoma* . Red and green in (a–c) indicate higher abundances in *T. leptosoma* and *T. longianguis* , respectively. Fit of the neutral community model (NCM) of community assembly in *T. leptosoma* (d) and *T. longianguis* (e). The solid line indicates the best fit to the NCM, and the dashed lines indicate 95% confidence intervals of the model prediction. The green and red circles represent OTUs with greater and lower frequencies than the prediction by NCM, respectively. Nm: Metacommunity size times immigration. *R* ^2^: The fit to the NCM.

To confirm the difference in food preference, the gut microbiota was characterized in 13 samples of both *T. longianguis* and *T. leptosoma* from Sunmcuo Lake, which yielded 3,634,154 sequences. After data processing and quality control, 8667 unique OTUs were generated, which were classified into 667 groups at the genus level belonging to 33 phyla. Similar taxonomic compositions were observed for *T. longianguis* and *T. leptosoma* , and Cyanobacteria and Proteobacteria were the predominant phyla in both species, with a relatively high abundance of Actinobacteria in *T. leptosoma* and a relatively high abundance of Firmicutes in *T. longianguis* (Figure 6a and Table S5). At the genus level, *Synechococcus* was the most abundant bacteria in both species, and *Rhodoplanes* and *Paulinella* accounted for greater percentages of *T. leptosoma* , whereas *Ruminococcus* and *Clostridium* were more abundant in *T. longianguis* (Figure 6b and Table S6). Comparisons of alpha diversity revealed that the Simpson and Pielou indices were greater in *T. leptosoma* than in *T. longianguis* , suggesting that the gut bacteria of the two *Triplophysa* fishes in Sunmcuo Lake significantly differed (Figure 6c,d). Based on Bray‐Curtis distances, the gut microbiota structure significantly differed between *T. longianguis* and *T. leptosoma* at the species, genus, and family levels (PERMANOVA, *p <* 0.05) but not at the OTU, order and phylum levels (Figure 6e and Table 4). LEfSe analysis revealed that 24 species of the four phyla presented distinct abundances in the bacterial communities of *T. leptosoma* compared with those in *T. longianguis* . The abundances of Bacteroidales in Bacteroidetes, Actinomyces and C111 in Acidimicrobiia, and unclassified species and SC_I_84 in Betaproteobacteria were greater in *T. leptosoma*, and the abundance of the genus *Ruminococcus* in the phylum Firmicutes was greater in *T. longianguis* (Figure 7a,b and Table S7). The functional prediction suggested that the different bacterial communities might significantly influence fatty acid salvage in their hosts (Figure 7c). With low R^2^ values of 15.2% and 21.6%, the occurrence frequency of intestinal bacterial OTUs fit weakly to the Sloan neutral model in *T. leptosoma* and *T. longianguis* , suggesting that deterministic processes, for example, ecological selection, such as diet, might play an important role in microbial community assembly in *T. leptosoma* and *T. longianguis* (Figure 7d,e). Both PERMANOVA and Sloan neutral model suggested that the divergence in diet selection might have contributed to the differences in the composition and function of the gut microbiota, which further supported the hypothesis that the sympatric speciation between *T. leptosoma* and *T. longianguis* was driven by food preferences.

**TABLE 4:** Summary of PERMANOVA results.

Level	Number	Mean of *R* ^2^	Mean of *F*	*p* value
OTU	220	0.060	1.65	0.065
Species	159	0.071	1.98	0.044
Genus	138	0.072	2.01	0.042
Family	117	0.070	1.96	0.049
Order	92	0.071	2.00	0.047
Class	52	0.068	1.88	0.061
Phylum	23	0.078	2.19	0.065

## 4 Discussion

### 4.1 Origin of *T. longianguis* Through Sympatric Speciation

*Triplophysa leptosoma* is widely distributed on the QTP, including Sunmcuo Lake, the Yellow River, the Lake Qinghai Basin, the Changjiang River, the Chaidamu River, and the Heixi River, whereas *T. longianguis* is restricted to Sunmcuo Lake, a semi‐enclosed lake that connects with the Yellow River through a small creek. The molecular phylogeny revealed the strong phylogeographic structure of *T. leptosoma* , with a monophyletic group of *T. leptosoma* and *T. longianguis* in Sunmcuo Lake. This phylogenetic structure implied that genetic differentiation within the geographic population of *T. leptosoma* might have occurred before the separation of *T. leptosoma* and *T. longianguis* in Sunmcuo Lake, which is consistent with estimated divergence times between *T. leptosoma* and *T. longianguis* (~3.94 Mya) as well as lineages between Sunmcuo Lake and the Yellow River (~7.49 Mya). Geological evidence has indicated that the formation of Sunmcuo Lake occurred after glacial retreat on the QTP (Lei et al. 2008). The Yellow River reached the present headwaters in the Zhaling‐Eling Basin until the Holocene (~0.03 Mya), suggesting that Sunmcuo Lake was maintained as an isolated inland lake throughout the late Pleistocene (Li et al. 2001). Therefore, the split between *T. leptosoma* and *T. longianguis* occurred in Sunmcuo Lake before it opened to the Yellow River. The phylogeny and divergence time reflected the development of historical drainage and supported the hypothesis that the sympatric speciation of *T. longianguis* from *T. leptosoma* in Sunmcuo Lake.

### 4.2 Trophic Partitioning in the Sympatric Speciation of *T. longianguis* and *T. leptosoma*

How divergence is achieved is the fundamental issue in sympatric speciation (Foote 2018). Compelling evidence suggests that feeding substrate preferences might facilitate trophic segregation and promote sympatric speciation through dietary niche partitioning (Zhao et al. 2009; Sun et al. 2022; Ramellini et al. 2024). In the present study, we detected a significant difference in the δ^13^C value between *T. longianguis* and *T. leptosoma* . Carbon stable isotopes are considered markers for feeding‐habitat axes (e.g., pelagic vs. benthic, shallow vs. deep) (Iken et al. 2001; Ñacari et al. 2023). Therefore, the lower *δ* ^13^C signature indicated the preference of *T. longianguis* to forage with phytoplankton, whereas the higher *δ* ^13^C value suggested the preference of *T. leptosoma* for benthic organisms, which was consistent with the examination of the gut content in the two coexistent Tibetan loaches. Both plants and algae were predominant in the food of *T. longianguis* and *T. leptosoma* , which might explain the lack of significant difference in the isotope analysis. Insects and zoobenthos were observed in the gut contents of *T. leptosoma* and were rarely identified in *T. longianguis* , which might result in higher *δ* ^13^C values in *T. leptosoma* . Species‐specific diets and stable isotopic signatures provide evidence of ecological isolation between two species, which reduces competition in coexistent species. Many studies have highlighted the importance of dietary niche partitioning in the divergence of coexisting species by avoiding interspecific competition for limited food resources (Correa and Winemiller 2014; Pillay et al. 2025). The sympatric speciation of *Gymnocypris eckloni scoliostomus* and *G. eckloni eckloni* in Sunmcuo Lake has been demonstrated to be driven by changes in their utilization of food resources (Zhao et al. 2009; Sun et al. 2022). The cichlid fish that coexist in Barombi Mbo crater Lake overlap in their dietary niches with the unique food resources of some species, which might contribute to adaptive radiation in sympatry (Galvez et al. 2022). These phenomena were consistent with our observations in *T. longianguis* and *T. leptosoma* , suggesting that the divergence in diet preferences might facilitate the sympatric speciation of *T. longianguis* and *T. leptosoma* . The current study identified evident differences in trophic morphology (e.g., mouth and lip structures) between *T. longianguis* and *T. leptosoma* , which was consistent with the distinct dietary niches inferred from stable isotope and gut content analyses. While these morphological variations likely contribute to diet partitioning, future studies integrating genome‐wide analysis, biomechanical modeling, and common‐garden feeding experiments will be needed to directly establish the causal links between trophic structures and food selection.

### 4.3 Synergistic Mechanism of Dietary Specialization via the Gut Microbiomes

**FIGURE 8:**
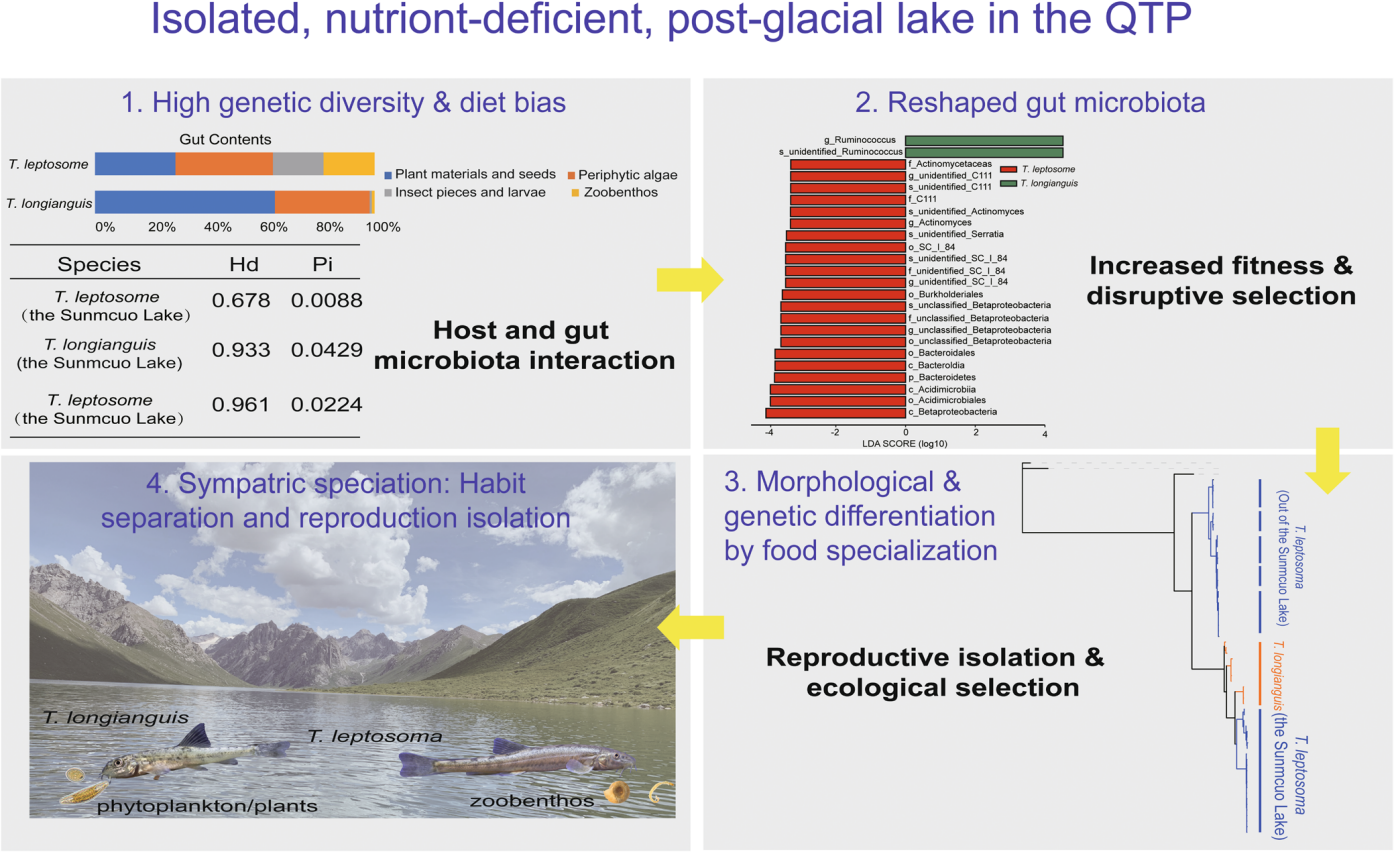
Conceptual diagram of the model of sympatric speciation in *T. leptosoma* and *T. longianguis* . The background photo was taken in Sunmcuo Lake by Dr. Kai Zhao (co‐author).

The gut microbiomes influence host diet selection by mediating the metabolism of essential nutrients, alleviating food competition among coexistent species (Zhang et al. 2013; Perofsky et al. 2019; Trevelline and Kohl 2022; Yang et al. 2025). Comparisons between two sympatric blind mole‐rats revealed no difference in community composition but rather variations in the functions of gut microorganisms, revealing the correspondence of the intestinal microbiota with ecological dietary factors in sympatric speciation (Kuang et al. 2022). Additionally, significant differences in the structure and diversity of the gut microbiota were observed in two sympatric rodents, with alterations in dietary adaptation and carbohydrate metabolism (Ren et al. 2025). In the present study, we also observed a similar composition of intestinal microorganisms, which was attributed to the overlapping food compositions and habits between *T. longianguis* and *T. leptosoma* . Firmicutes are generally more abundant in herbivores, and the genus *Ruminococcus* in the phylum Firmicutes contributes to cellulose degradation (Cann et al. 2016). *Triplophysa longianguis* might benefit from the greater abundance of Firmicutes in the gut for the better digestion of the higher levels of plant materials and seeds in their food. The abundance of Bacteroides, Actinomyces and Betaproteobacteria, which are generally high in omnivores, is usually related to adaptation to diverse diets, with the ability to decompose proteins and carbohydrates, such as polysaccharides and chitin (Pepper and Gentry 2015). The relatively high abundance of these bacteria might contribute to the metabolism of insects and zoobenthos, the unique food resources of *T. leptosoma* . This result indicated the reciprocal influence of dietary specialization on gut microbiota assembly and function in that species‐abundant intestinal bacteria might further promote trophic specialization. Therefore, we propose that the ancestral population of *T. longianguis* and *T. leptosoma* likely harbored substantial genetic and morphological diversity (Figure 8). Upon isolation in Sunmcuo Lake, the limited food resources placed strong ecological pressure on the ancestral population and favored disruptive selection on heritable variation in trophic morphology (e.g., lip structure) and food choice. This diet bias led to population divergence, resulting in the formation of two lineages that specialize in phytoplankton/plants and benthic organisms. The different food resources further imposed selection on the gut microbiota composition, which promoted digestive efficiency and increased host fitness. This positive effect of the gut microbiomes on the host reinforced diet specialization, which in turn strengthened the divergence of the gut microbiota community. This host–gut microbiome interaction facilitated habitat partitioning and genetic differentiation and ultimately led to reproductive isolation and sympatric speciation of *T. longianguis* and *T. leptosoma* . Therefore, our findings suggest that the amplification effect of gut microbiota differentiation accelerated ecological and reproductive isolation, which promoted sympatric speciation of *T. longianguis* and *T. leptosoma* , which was initially mediated by diet bias. This synergy between the gut microbiota and diet highlights the involvement of multiple factors in sympatric speciation in the resource‐limited environment of the QTP.

### 4.4 Phylogenetics of *Triplophysa* Fishes

The taxonomy of the genus *Triplophysa* has long been debated. *Hedinichthys* was classified as a subgenus of the genus *Triplophysa* in previous studies (Zhu 1989; Wu and Wu 1992) but was defined as an independent genus by He et al. (2024). The phylogenetic tree of the mitogenome revealed the monophyly of *Triplophysa* fish with clear separation from *H. yarkandensis*, which supported the taxonomic revision of *Triplophysa* and *Hedinichthys* as two genera. The genus *Triplophysa* is classified into four lineages, among which cave‐dwelling loaches are grouped into clade A, which is indicative of the potential adaptive mechanism of subterranean ecosystems. The phylogeography of *Triplophysa* on the QTP reportedly resulted from the occurrence of geological events during the uplift of the QTP (Wang et al. 2016; Jin et al. 2022). The geographic distribution of *Triplophysa* fishes in Clades B1, B2 and B3 was consistent with previous reports based on whole‐genome sequencing and mitochondrial genes, supporting the phased uplift process of the QTP (Wang et al. 2016; Feng, Zhou, et al. 2019; Qian et al. 2023). The geographic pattern of *Triplophysa* fish also indicated that allopatric speciation was the major speciation mode of the QTP. The paraphyly of *T. leptosoma* geographic populations might have resulted from population differentiation due to allopatric isolation. Geographic isolation is considered one of the major driving forces of population divergence among endemic fish on the QTP. A series of studies revealed that vicariance caused by the uplift of the QTP led to population differentiation in *Triplophysa tenuis* and *Triplophysa robusta* (Feng, Zhou, et al. 2019; Feng et al. 2026). Additionally, convergent evolution has been reported to mislead the taxonomic positions of geographic lineages in *Triplophysa stoliczkae* (Feng, Tang, et al. 2019). Multiple paraphyletic clades of *T. leptosoma* might be attributed to the phenotypic convergence of different ancestral species that evolved under the same eco‐environmental pressure on Sunmcuo Lake. Additional samples from all the populations might reveal the evolutionary modes of *T. leptosoma* .

## 5 Conclusion

In conclusion, our results confirmed that *T. longianguis* and *T. leptosoma* in Sunmcuo Lake originated through sympatric speciation, which was driven mainly by dietary niche partitioning as the key ecological factor. The differences in the composition and function of the gut microbiomes are consistent with diet specialization, which contributes to host adaptation to different diet niches. These findings expand our knowledge of the synergistic effects of dietary partitioning and the gut microbiomes on sympatric speciation and provide valuable insights into the formation and maintenance of fish biodiversity on the QTP. Further analyses are needed to elucidate the genomic basis of food specialization and its relationship with reproductive isolation.

## Author Contributions

**Sijia Liu:** formal analysis (lead), funding acquisition (supporting), investigation (lead), writing – original draft (equal), writing – review and editing (supporting). **Ruoyu Zhang:** formal analysis (equal), investigation (equal), writing – original draft (equal), writing – review and editing (equal). **Zhengli Wang:** formal analysis (supporting), investigation (supporting), writing – review and editing (supporting). **Kemao Li:** data curation (equal), investigation (supporting), writing – review and editing (supporting). **Wulong Ma:** data curation (supporting), investigation (supporting), writing – review and editing (supporting). **Jinliang Wei:** formal analysis (supporting), investigation (supporting), writing – review and editing (supporting). **Mingzhu Wang:** investigation (supporting), writing – review and editing (supporting). **Wenjun Ma:** formal analysis (supporting), writing – review and editing (supporting). **Shengxue Chen:** investigation (supporting), writing – review and editing (supporting). **Kai Zhao:** funding acquisition (equal), writing – review and editing (equal). **Guogang Li:** funding acquisition (equal), project administration (supporting), supervision (equal), writing – review and editing (equal). **Fei Tian:** conceptualization (equal), formal analysis (supporting), funding acquisition (supporting), project administration (equal), writing – original draft (lead), writing – review and editing (lead).

## Funding

This work was supported by the National Natural Science Foundation of China (No. 32071489), the Sino BON‐Inland Water Fish Diversity Observation Network, the Biological Resources Program (Chinese Academy of Sciences, No. CAS‐TAX‐24‐069), the 2023 Award Fund of Qinghai Provincial Key Laboratory of Animal Ecological Genomics (QHEG‐2023‐01), and the Youth Fund of Qinghai University (2024‐QNY‐6).

## Ethics Statement

All animal procedures were approved by the Animal Care and Use Committee of the Northwest Institute of Plateau Biology, Chinese Academy of Sciences (Approval No. NWIPB‐202006).

## Consent

The authors have nothing to report.

## Conflicts of Interest

The authors declare no conflicts of interest.

## Supporting information


**Table S1:** Sampling sites.
**Table S2:** Mitochondrial genomes used in the study.
**Table S3:** Haplotypes of Cyt *b* used in the study.
**Table S4:** Sequencing statistics of gut microbiomes.
**Table S5:** Bacteria compositon at phylum level.
**Table S6:** Bacteria compositon at genus level.
**Table S7:** LEfSe result of bacteria with significant differences between *T. leptosome* and *T. longianguis* .

**Figure S1:** Examples of microscopic examination of gut contents in *T. longianguis* and *T. leptosoma* . (a) and (b) were zoobenthos; (c) and (d) were zooplankton; (e) and (f) were phytoplankton.

## Data Availability

The mitogenomes of *T. longianguis* and *T. leptosoma* were deposited in NCBI under the accession numbers PX596372 and PX596373. The raw 16S rRNA gene sequencing reads were deposited in the NCBI SRA under accession numbers SAMN53422679–SAMN53422706.

## References

[ece374001-bib-0001] Barluenga, M. , K. N. Stölting , W. Salzburger , M. Muschick , and A. Meyer . 2006. “Sympatric Speciation in Nicaraguan Crater Lake Cichlid Fish.” Nature 439: 719–723. 16467837 10.1038/nature04325

[ece374001-bib-0002] Bernt, M. , A. Donath , F. Jühling , et al. 2013. “MITOS: Improved De Novo Metazoan Mitochondrial Genome Annotation.” Molecular Phylogenetics and Evolution 69: 313–319. 22982435 10.1016/j.ympev.2012.08.023

[ece374001-bib-0003] Bird, C. E. , I. Fernandez‐Silva , D. J. Skillings , and R. J. Toonen . 2012. “Sympatric Speciation in the Post “Modern Synthesis” Era of Evolutionary Biology.” Evolutionary Biology 39: 158–180.

[ece374001-bib-0004] Bokulich, N. A. , B. D. Kaehler , J. R. Rideout , et al. 2018. “Optimizing Taxonomic Classification of Marker‐Gene Amplicon Sequences With QIIME 2's q2‐Feature‐Classifier Plugin.” Microbiome 6: 90. 29773078 10.1186/s40168-018-0470-z PMC5956843

[ece374001-bib-0005] Bolnick, D. I. , and B. M. Fitzpatrick . 2007. “Sympatric Speciation: Models and Empirical Evidence.” Annual Review of Ecology, Evolution, and Systematics 38: 459–487.

[ece374001-bib-0006] Bolyen, E. , J. R. Rideout , M. R. Dillon , et al. 2019. “Reproducible, Interactive, Scalable and Extensible Microbiome Data Science Using QIIME 2.” Nature Biotechnology 37: 852–857. 10.1038/s41587-019-0209-9 PMC7015180 31341288

[ece374001-bib-0007] Cann, I. , R. C. Bernardi , and R. I. Mackie . 2016. “Cellulose Degradation in the Human Gut: *Ruminococcus champanellensis* Expands the Cellulosome Paradigm.” Environmental Microbiology 18: 307–310. 26781441 10.1111/1462-2920.13152

[ece374001-bib-0008] Chen, W. , K. Ren , A. Isabwe , H. Chen , M. Liu , and J. Yang . 2019. “Stochastic Processes Shape Microeukaryotic Community Assembly in a Subtropical River Across Wet and Dry Seasons.” Microbiome 7: 138. 31640783 10.1186/s40168-019-0749-8 PMC6806580

[ece374001-bib-0009] Chen, Y. , Y. Chen , and H. Liu . 1996. “Studies on the Position of the Qinghai‐Xizang Plateau Region in Zoogeographic Divisions and Its Eastern Demarcation Line.” Acta Hydrobiologica Sinica 20: 97–103.

[ece374001-bib-0010] Correa, S. B. , and K. O. Winemiller . 2014. “Niche Partitioning Among Frugivorous Fishes in Response to Fluctuating Resources in the Amazonian Floodplain Forest.” Ecology 95: 210–224. 24649660 10.1890/13-0393.1

[ece374001-bib-0011] Coyne, J. A. , and H. A. Orr . 2004. Speciation. Sinauer Associates.

[ece374001-bib-0012] De León, L. F. , J. Podos , T. Gardezi , A. Herrel , and A. P. Hendry . 2014. “Darwin's Finches and Their Diet Niches: The Sympatric Coexistence of Imperfect Generalists.” Journal of Evolutionary Biology 27: 1093–1104. 24750315 10.1111/jeb.12383

[ece374001-bib-0013] Degregori, S. , N. M. D. Schiettekatte , J. M. Casey , et al. 2024. “Host Diet Drives Gut Microbiome Convergence Between Coral Reef Fishes and Mammals.” Molecular Ecology 33: e17520. 39205506 10.1111/mec.17520

[ece374001-bib-0014] Dixon, P. 2003. “VEGAN, a Package of R Functions for Community Ecology.” Journal of Vegetation Science 14: 927–930.

[ece374001-bib-0015] Elmer, K. R. , T. K. Lehtonen , A. F. Kautt , C. Harrod , and A. Meyer . 2010. “Rapid Sympatric Ecological Differentiation of Crater Lake Cichlid Fishes Within Historic Times.” BMC Biology 8: 60. 20459869 10.1186/1741-7007-8-60 PMC2880021

[ece374001-bib-0016] Emmanuel, P. , S. Klaus , and S. Russell . 2018. “Ape 5.0: An Environment for Modern Phylogenetics and Evolutionary Analyses in R.” Bioinformatics 35: 526–528. 10.1093/bioinformatics/bty633 30016406

[ece374001-bib-0017] Feng, C. , Y. Tang , S. Liu , F. Tian , C. Zhang , and K. Zhao . 2019. “Multiple Convergent Events Created a Nominal Widespread Species: Triplophysa Stoliczkae (Steindachner, 1866) (Cobitoidea: Nemacheilidae).” BMC Evolutionary Biology 19: 177. 31484504 10.1186/s12862-019-1503-3 PMC6724303

[ece374001-bib-0018] Feng, C. , Y. Wu , F. Tian , et al. 2017. “Elevational Diversity Gradients of Tibetan Loaches: The Relative Roles of Ecological and Evolutionary Processes.” Ecology and Evolution 7: 9970–9977. 29238529 10.1002/ece3.3504 PMC5723583

[ece374001-bib-0019] Feng, C. , R. Zhang , C. Tong , et al. 2026. “Phylogenomic Analysis Reveals the Demographic History and Cryptic Barriers of Three Desert Fishes.” Journal of Systematics and Evolution 64: 66–76.

[ece374001-bib-0020] Feng, C. , W. Zhou , Y. Tang , et al. 2019. “Molecular Systematics of the *Triplophysa robusta* (Cobitoidea) Complex: Extensive Gene Flow in a Depauperate Lineage.” Molecular Phylogenetics and Evolution 132: 275–283. 30550962 10.1016/j.ympev.2018.12.009

[ece374001-bib-0021] Foote, A. D. 2018. “Sympatric Speciation in the Genomic Era.” Trends in Ecology & Evolution 33: 85–95. 29198471 10.1016/j.tree.2017.11.003

[ece374001-bib-0022] Galvez, J. R. , M. E. St. John , K. McLean , C. Dening Touokong , L. N. Gonwouo , and C. H. Martin . 2022. “Trophic Specialization on Unique Resources Despite Limited Niche Divergence in a Celebrated Example of Sympatric Speciation.” Ecology of Freshwater Fish 31: 675–692. 36211622 10.1111/eff.12661 PMC9542214

[ece374001-bib-0023] Gavrilets, S. , and A. Vose . 2007. “Case Studies and Mathematical Models of Ecological Speciation. 2. Palms on an Oceanic Island.” Molecular Ecology 16: 2910–2921. 17614906 10.1111/j.1365-294X.2007.03304.x

[ece374001-bib-0024] Getz, W. M. , R. Salter , D. P. Seidel , and P. van Hooft . 2016. “Sympatric Speciation in Structureless Environments.” BMC Evolutionary Biology 16: 50. 26922946 10.1186/s12862-016-0617-0 PMC4770699

[ece374001-bib-0025] Grant, B. R. , and P. R. Grant . 1979. “Darwin's Finches: Population Variation and Sympatric Speciation.” Proceedings of the National Academy of Sciences 76: 2359–2363. 10.1073/pnas.76.5.2359 PMC383600 16592654

[ece374001-bib-0026] Greene, L. K. , C. V. Williams , R. E. Junge , et al. 2020. “A Role for Gut Microbiota in Host Niche Differentiation.” ISME Journal 14: 1675–1687. 32238913 10.1038/s41396-020-0640-4 PMC7305313

[ece374001-bib-0027] Greiner, S. , P. Lehwark , and R. Bock . 2019. “OrganellarGenomeDRAW (OGDRAW) Version 1.3.1: Expanded Toolkit for the Graphical Visualization of Organellar Genomes.” Nucleic Acids Research 47: W59–W64. 30949694 10.1093/nar/gkz238 PMC6602502

[ece374001-bib-0028] He, S. P. , S. Q. Zhu , R. H. Ding , L. D. Yang , and W. X. Cao . 2024. “Nemacheilinae.” In Fauna Sinica Osteichthyes Cypriniformes I. Since Press.

[ece374001-bib-0066] Herzenstein, S. 1888. Fische. Eggers & Company.

[ece374001-bib-0029] Iken, K. , T. Brey , U. Wand , J. Voigt , and P. Junghans . 2001. “Food Web Structure of the Benthic Community at the Porcupine Abyssal Plain (NE Atlantic): A Stable Isotope Analysis.” Progress in Oceanography 50: 383–405.

[ece374001-bib-0030] Jin, L. , Z. Li , C. Wang , et al. 2022. “Contrasting Population Differentiation in Two Sympatric Triplophysa Loaches on the Qinghai‐Tibet Plateau.” Frontiers in Genetics 13: 958076. 36092882 10.3389/fgene.2022.958076 PMC9452750

[ece374001-bib-0031] Katoh, K. , K. Misawa , K. Kuma , and T. Miyata . 2002. “MAFFT: A Novel Method for Rapid Multiple Sequence Alignment Based on Fast Fourier Transform.” Nucleic Acids Research 30: 3059–3066. 12136088 10.1093/nar/gkf436 PMC135756

[ece374001-bib-0032] Kuang, Z. , F. Li , Q. Duan , C. Tian , E. Nevo , and K. Li . 2022. “Host Diet Shapes Functionally Differentiated Gut Microbiomes in Sympatric Speciation of Blind Mole Rats in Upper Galilee, Israel.” Frontiers in Microbiology 13: 1062763. 36458196 10.3389/fmicb.2022.1062763 PMC9707624

[ece374001-bib-0033] Kumar, S. , M. Suleski , J. M. Craig , et al. 2022. “TimeTree 5: An Expanded Resource for Species Divergence Times.” Molecular Biology and Evolution 39: msac174. 35932227 10.1093/molbev/msac174 PMC9400175

[ece374001-bib-0034] Langille, M. G. , J. Zaneveld , J. G. Caporaso , et al. 2013. “Predictive Functional Profiling of Microbial Communities Using 16S rRNA Marker Gene Sequences.” Nature Biotechnology 31: 814–821. 10.1038/nbt.2676 PMC3819121 23975157

[ece374001-bib-0035] Lei, Y. B. , H. C. Zhang , H. M. Shang , L. Q. Yang , G. L. Lei , and W. X. Zhang . 2008. “Lake Evolution and Glaciation of the Nianbaoyuze Mountain in the Tibetan Plateau Since the Middle of the Last Glacial.” Quaternary Sciences 28: 132–139.

[ece374001-bib-0074] Letourneur, Y. , T. Lison de Loma , P. Richard , et al. 2013. “Identifying Carbon Sources and Trophic Position of Coral Reef Fishes Using Diet and Stable Isotope (δ15N and δ13C) Analyses in two Contrasted Bays in Moorea, French Polynesia.” Coral Reefs 32: 1091–1102.

[ece374001-bib-0069] Li, J. B. , X. Z. Wang , X. H. Kong , K. Zhao , S. P. He , and R. L. Mayden . 2008. “Variation Patterns of the Mitochondrial 16S rRNA Gene With Secondary Structure Constraints and Their Application to Phylogeny of Cyprinine Fishes (Teleostei: Cypriniformes).” Molecular Phylogenetics and Evolution 47: 472–487. 18378468 10.1016/j.ympev.2007.09.012

[ece374001-bib-0036] Li, J. J. , X. M. Fang , and H. Z. Ma . 2001. “Late Cenozoic Intensive Uplift of Qinghai‐Xizang Plateau and Its Impacts on Environments in Surrounding Area.” Quaternary Sciences 21, no. 5: 381–391.

[ece374001-bib-0071] Librado, P. , and J. Rozas . 2009. “DnaSP v5: A Software for Comprehensive Analysis of DNA Polymorphism Data.” Bioinformatics 25: 1451–1452. 19346325 10.1093/bioinformatics/btp187

[ece374001-bib-0076] Martin, M. 2011. “Cutadapt Removes Adapter Sequences from High‐Throughput Sequencing Reads.” EMBnet. journal 17: 10–12.

[ece374001-bib-0037] Ñacari, L. A. , R. Escribano , C. Harrod , and M. E. Oliva . 2023. “Combined Use of Carbon, Nitrogen and Sulfur Stable Isotopes Reveal Trophic Structure and Connections in Deep‐Sea Mesopelagic and Demersal Fish Communities From the Southeastern Pacific Ocean.” Deep Sea Research Part I: Oceanographic Research Papers 197: 104069.

[ece374001-bib-0038] Neves, M. P. , P. Kratina , C. B. Fialho , K. M. Evans , and R. L. Delariva . 2025. “Morphological Divergence and the Complexity of Trophic Niche Plasticity of Tetra Fishes in Subtropical Streams.” Hydrobiologia 853: 1249–1271.

[ece374001-bib-0039] Nosil, P. , D. J. Funk , and D. Ortiz‐Barrientos . 2009. “Divergent Selection and Heterogeneous Genomic Divergence.” Molecular Ecology 18: 375–402. 19143936 10.1111/j.1365-294X.2008.03946.x

[ece374001-bib-0040] Pepper, I. L. , and T. J. Gentry . 2015. “Chapter 4—Earth Environments.” In Environmental Microbiology, edited by I. L. Pepper , C. P. Gerba , and T. J. Gentry , Third ed., 59–88. Academic Press.

[ece374001-bib-0041] Perofsky, A. C. , R. J. Lewis , and L. A. Meyers . 2019. “Terrestriality and Bacterial Transfer: A Comparative Study of Gut Microbiomes in Sympatric Malagasy Mammals.” ISME Journal 13: 50–63. 30108305 10.1038/s41396-018-0251-5 PMC6299109

[ece374001-bib-0042] Pillay, K. , S. Creer , A. M. Tyers , et al. 2025. “Dietary Differentiation Between Sympatric Ecotypes of *Astatotilapia calliptera* From Lake Masoko (Kisiba), Tanzania Revealed by Metabarcoding.” Environmental DNA 7: e70146.

[ece374001-bib-0043] Price, M. N. , P. S. Dehal , and A. P. Arkin . 2009. “FastTree: Computing Large Minimum Evolution Trees With Profiles Instead of a Distance Matrix.” Molecular Biology and Evolution 26: 1641–1650. 19377059 10.1093/molbev/msp077 PMC2693737

[ece374001-bib-0044] Qian, Y. , M. Meng , C. Zhou , et al. 2023. “The Role of Introgression During the Radiation of Endemic Fishes Adapted to Living at Extreme Altitudes in the Tibetan Plateau.” Molecular Biology and Evolution 40: masd129. 10.1093/molbev/msad129 PMC10297026 37247387

[ece374001-bib-0045] Quast, C. , E. Pruesse , P. Yilmaz , et al. 2013. “The SILVA Ribosomal RNA Gene Database Project: Improved Data Processing and Web‐Based Tools.” Nucleic Acids Research 41: D590–D596. 23193283 10.1093/nar/gks1219 PMC3531112

[ece374001-bib-0073] Rambaut, A. , A. J. Drummond , D. Xie , G. Baele , and M. A. Suchard . 2018. “Posterior Summarization in Bayesian Phylogenetics Using Tracer 1.7.” Systematic Biology 67: 901–904. 29718447 10.1093/sysbio/syy032 PMC6101584

[ece374001-bib-0046] Ramellini, S. , E. Crepet , S. Lapadula , and A. Romano . 2024. “Trophic Niche Segregation in a Guild of Top Predators Within the Mediterranean Basin.” Current Zoology 70: 697–706. 39678818 10.1093/cz/zoae001 PMC11634680

[ece374001-bib-0047] Recuerda, M. , M. Palacios , O. Frias , et al. 2023. “Adaptive Phenotypic and Genomic Divergence in the Common Chaffinch ( *Fringilla coelebs* ) Following Niche Expansion Within a Small Oceanic Island.” Journal of Evolutionary Biology 36: 1226–1241. 37485603 10.1111/jeb.14200

[ece374001-bib-0048] Ren, Y. , M. Tao , G. Guo , et al. 2025. “Gut Microbiota Provide co‐Existing Strategies for Two Species of Symmetrically Distributed Rodents in Competition for Food.” Ecology and Evolution 15: e72290. 41122693 10.1002/ece3.72290 PMC12536269

[ece374001-bib-0049] Rennison, D. J. , S. M. Rudman , and D. Schluter . 2019. “Parallel Changes in Gut Microbiome Composition and Function During Colonization, Local Adaptation and Ecological Speciation.” Proceedings of the Royal Society B: Biological Sciences 286: 1911. 10.1098/rspb.2019.1911 PMC6939261 31795865

[ece374001-bib-0050] Richards, E. J. , M. R. Servedio , and C. H. Martin . 2019. “Searching for Sympatric Speciation in the Genomic Era.” BioEssays 41: e1900047. 31245871 10.1002/bies.201900047 PMC8175013

[ece374001-bib-0075] Rognes, T. , T. Flouri , B. Nichols , C. Quince , and F. Mahé . 2016. “VSEARCH: A Versatile Open Source Tool for Metagenomics.” PeerJ 4. 10.7717/peerj.2584 PMC5075697 27781170

[ece374001-bib-0051] Segata, N. , J. Izard , L. Waldron , et al. 2011. “Metagenomic Biomarker Discovery and Explanation.” Genome Biology 12: R60. 21702898 10.1186/gb-2011-12-6-r60 PMC3218848

[ece374001-bib-0052] Sloan, W. T. , M. Lunn , S. Woodcock , I. M. Head , S. Nee , and T. P. Curtis . 2006. “Quantifying the Roles of Immigration and Chance in Shaping Prokaryote Community Structure.” Environmental Microbiology 8: 732–740. 16584484 10.1111/j.1462-2920.2005.00956.x

[ece374001-bib-0072] Suchard, M. A. , P. Lemey , G. Baele , D. L. Ayres , A. J. Drummond , and A. Rambaut . 2018. “Bayesian Phylogenetic and Phylodynamic Data Integration Using BEAST 1.10. Virus Evol 4.” 10.1093/ve/vey016 PMC6007674 29942656

[ece374001-bib-0053] Sun, N. , L. Yang , F. Tian , et al. 2022. “Sympatric or Micro‐Allopatric Speciation in a Glacial Lake? Genomic Islands Support Neither.” National Science Review 9: nwac291. 36778108 10.1093/nsr/nwac291 PMC9905650

[ece374001-bib-0054] Sutra, N. , J. Kusumi , J. Montenegro , et al. 2019. “Evidence for Sympatric Speciation in a Wallacean Ancient Lake.” Evolution 73: 1898–1915. 31407798 10.1111/evo.13821

[ece374001-bib-0055] Tamura, K. , G. Stecher , and S. Kumar . 2021. “MEGA11: Molecular Evolutionary Genetics Analysis Version 11.” Molecular Biology and Evolution 38: 3022–3027. 33892491 10.1093/molbev/msab120 PMC8233496

[ece374001-bib-0070] Thompson, J. D. , D. G. Higgins , and T. J. Gibson . 1994. “CLUSTAL W: Improving the Sensitivity of Progressive Multiple Sequence Alignment Through Sequence Weighting, Position‐Specific Gap Penalties and Weight Matrix Choice.” Nucleic Acids Research 22: 4673–4680. 7984417 10.1093/nar/22.22.4673 PMC308517

[ece374001-bib-0056] Todd Streelman, J. , and P. D. Danley . 2003. “The Stages of Vertebrate Evolutionary Radiation.” Trends in Ecology & Evolution 18: 126–131.

[ece374001-bib-0057] Trevelline, B. K. , and K. D. Kohl . 2022. “The Gut Microbiome Influences Host Diet Selection Behavior.” Proceedings of the National Academy of Sciences of the United States of America 119: e2117537119. 35439064 10.1073/pnas.2117537119 PMC9169907

[ece374001-bib-0058] Wang, Y. , Y. Shen , C. Feng , et al. 2016. “Mitogenomic Perspectives on the Origin of Tibetan Loaches and Their Adaptation to High Altitude.” Scientific Reports 6: 29690. 27417983 10.1038/srep29690 PMC4945904

[ece374001-bib-0059] Wu, Y. , and C. Wu . 1992. The Fishes of the Qinghai‐Xizang Plateau. Sichuan Publishing House of Science and Technology.

[ece374001-bib-0067] Wu, Y. F. , and C. Z. Wu . 1984. “Notes on Fishes from Lake Sunm Cuo of Qinghai Province, China.” Acta Zootaxonomica Sinica 9: 326–329.

[ece374001-bib-0068] Xiao, W. , Y. Zhang , and H. Liu . 2001. “Molecular Systematics of Xenocyprinae (Teleostei: Cyprinidae): Taxonomy, Biogeography, and Coevolution of a Special Group Restricted in East Asia.” Molecular Phylogenetics and Evolution 18: 163–173. 11161753 10.1006/mpev.2000.0879

[ece374001-bib-0060] Yang, X. , X. Wang , M. Zhang , et al. 2025. “Gut Mycobiota of Three Rhinopithecus Species Provide New Insights Into the Association Between Diet and Environment.” Integrative Zoology 20: 936–947. 39690132 10.1111/1749-4877.12932 PMC12463750

[ece374001-bib-0061] Yuan, D. , X. Chen , H. Gu , et al. 2020. “Chromosomal Genome of *Triplophysa bleekeri* Provides Insights Into Its Evolution and Environmental Adaptation.” GigaScience 9: giaa132. 33231676 10.1093/gigascience/giaa132 PMC7684707

[ece374001-bib-0062] Zhang, D. , F. Gao , I. Jakovlic , et al. 2020. “PhyloSuite: An Integrated and Scalable Desktop Platform for Streamlined Molecular Sequence Data Management and Evolutionary Phylogenetics Studies.” Molecular Ecology Resources 20: 348–355. 31599058 10.1111/1755-0998.13096

[ece374001-bib-0063] Zhang, Y.‐F. , L. Tong , W.‐H. Ji , and J.‐Q. Lu . 2013. “Comparison of Food Hoarding of Two Sympatric Rodent Species Under Interspecific Competition.” Behavioural Processes 92: 60–64. 23124017 10.1016/j.beproc.2012.10.012

[ece374001-bib-0064] Zhao, K. , Z. Y. Duan , Z. G. Peng , et al. 2009. “The Youngest Split in Sympatric Schizothoracine Fish (Cyprinidae) is Shaped by Ecological Adaptations in a Tibetan Plateau Glacier Lake.” Molecular Ecology 18: 3616–3628. 19674313 10.1111/j.1365-294X.2009.04274.x

[ece374001-bib-0065] Zhu, S. Q. 1989. Monograph on the Loaches of China. Jiangsu Science and Technonlogy Press.

