# Supplementary material for: Sympatric Speciation of Tibetan Loaches (Triplophysa) Driven by Dietary Niche Specialization
Source: Ecol Evol. 2026 Jul 22;16(7):e74001. doi: 10.1002/ece3.74001 (PMC13391240; doi:10.1002/ece3.74001)

Fig. S1 Examples of microscopic examination of gut contents in *T. longianguis* and *T. leptosoma*. (a) and (b) were zoobenthos; (c) and (d) were zooplankton; (e) and (f) were phytoplankton.


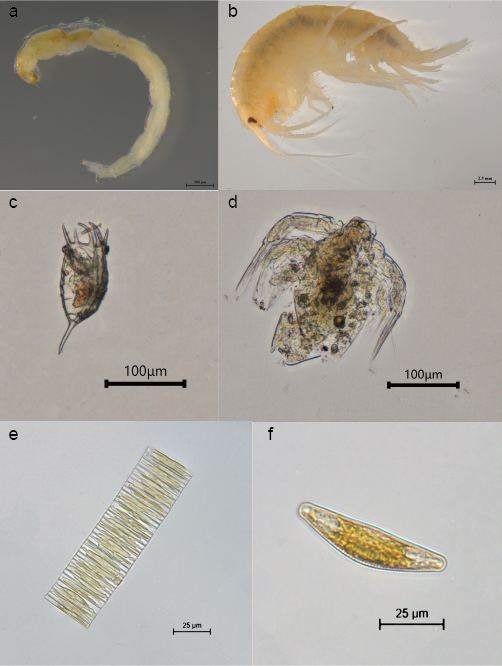

Supplement: Supplementary file 2 — Figure S1: Examples of microscopic examination of gut contents in T. longianguis and T. leptosoma . (a) and (b) were zoobenthos; (c) and (d) were zooplankton; (e) and (f) were phytoplankton. [file ECE3-16-e74001-s001.docx]
